# Alopecia and pet: a case report

**DOI:** 10.31744/einstein_journal/2022RC6881

**Published:** 2022-06-28

**Authors:** Eduardo Cukierman, Thiago Zinsly Sampaio Camargo, Laís Pereira Bueno Millan, Marianna Ribeiro de Menezes Freire, Lucas Franco Mendes Carneiro, Renata Dejtiar Waksman

**Affiliations:** 1 Hospital Israelita Albert Einstein São Paulo SP Brazil Hospital Israelita Albert Einstein, São Paulo, SP, Brazil.

**Keywords:** Tinea, Tinea capitis, Fungi, Scalp conditions

## Abstract

Dermatophytoses are fungal infections affecting the skin and cutaneous annexes. This clinical case report describes a 7-year-old girl with *Kerion celsi*, a severe manifestation of *Tinea capitis*. The patient presented with painful edematous crusty scalp lesions and alopecia, which required surgical debridement and long-term antifungal treatment. Culture of samples collected from scalp and arm skin lesions (patient and patient’s mother respectively) were positive for *Trichophyton mentagrophytes*. The family owned a pet guinea pig. This particular dermatophytosis is easily transmitted from guinea pigs to humans, with some studies showing up to 34.9% prevalence of *Trichophyton mentagrophytes* infection in these animals.

## INTRODUCTION

Dermatophytoses are fungal infections affecting primarily the skin, hair and nails. Dermatophytes are filamentous fungi belonging to three different genera: *Trichophyton, Microsporum* and *Epidermophyton.*^(1,2)^ The physiopathology of the disease includes keratin degradation, unbalanced keratin production and delayed hypersensitivity reaction.^(3)^

The infection is usually acquired from other human beings or animals, or through contact with contaminated objects.^(3,4)^ Clinical manifestations include skin scaling and hyperemia and are often diagnostic. However, diagnosis can be confirmed using the KOH test (potassium 10% hydroxide test) or fungal culture if needed.^(4,5)^

*Tinea capitis* (dermatophytosis of the scalp) is a prevalent disease in prepubertal children, which often manifests as focal areas of itching and alopecia with broken hair shafts, with or without cervical lymph node swelling. Generalized skin scaling and formation of edematous, crusty and painful plaques (*Kerion celsi*) may occur in more severe cases.^(4)^

*Kerion celsi* tends to develop in children aged 5 to 10 years and is caused primarily by Zoophyllic fungi such as *Microsporum canis, Trichophyton verrucosum, Trichophyton equinum* and *Microsporum nanum.* Although less common, Anthropophillic fungi like *Trichophyton mentagrophytes* may also cause *Kerion celsi*, which may progress to scarring alopecia in persistent cases.^(5)^

*Trichophyton mentagrophytes* is commonly found in animals increasingly seen in the household environment, such as rabbits, guinea pigs, porcupines, chinchillas and other rodents.^(6,7)^

## CASE REPORT

A 7-year-old female patient presented to the emergency department on November 20, 2019 with an extensive scalp lesion and unrelated fever. The lesion had been noticed 2 weeks back and had become worse over the last 7 days. She had a history of scabies and recurrent *Tinea corporis* and had been submitted to sequential treatment with cephalexin (7 days), clindamycin (5 days) and cefadroxil. Cefadroxil had been taken for 2 days only and was discontinued and replaced by intravenous antimicrobial medication upon admission. She had also been prescribed topical terbinafine for 7 days and isoconazole nitrate.

**Figure 1:**
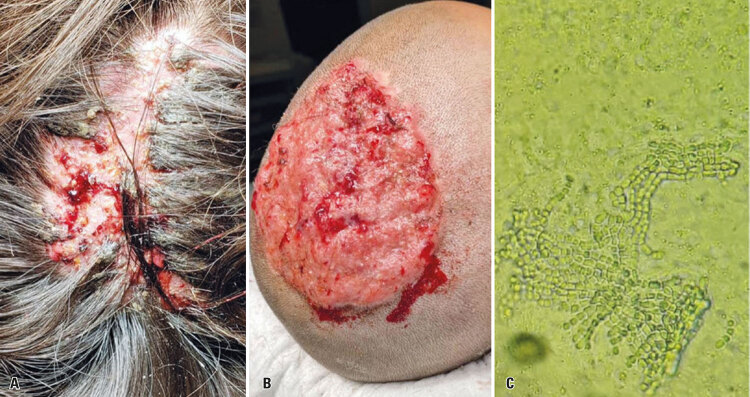
(A) Scalp lesion seen upon initial assessment in the emergency department; (B) Scalp lesion after surgical wound debridement; (C) Dermatophyte detected on direct examination

Initial physical examination revealed good general health and extensive scalp lesions occupying a 15 x 20cm area, particularly in frontoparietal regions. Lesions were accompanied by purulent discharge, an alopecic area measuring approximately 10 x 3cm, superficial skin swelling and severe pain (Figure 1A).

Laboratory tests revealed neutrophilic leukocytosis, ERS of 61mm and CRP levels <5. Serum biochemistry was unremarkable. Scalp pain and discomfort precluded sonographic assessment. Therefore, computed tomography (CT) of the head was requested to rule out fluid collections and/or abscess formation. Findings revealed extracranial frontoparietal soft tissue lesions in the right median and paramedian high convexity surfaces. These were non-specific and potentially inflammatory in nature. Underlying bone was apparently unaffected.

Laboratory and imaging findings, lack of response to topical and oral antifungal therapy, progressive deterioration of scalp lesions and signs of impending secondary infection justified hospital admission and prescription of intravenous antimicrobial therapy (sodium cefuroxime). Surgical intervention was also scheduled.

Surgery was performed on November 21, 2019. Surgical procedures consisted of wound debridement, drainage of fluid collections and tissue sampling for biopsy and culture, followed by application of an occlusive dressing (Figure 1B). Direct examination suggested dermaphytosis (Figure 1C). Therefore, combined treatment with oral terbinafine and intravenous fluconazole was prescribed.

Preliminary anatomopathological results obtained on November 23, 2019 showed fungal infection. Terbinafine was then replaced with griseofulvin and intravenous fluconazole maintained.

Final anatomopathological results (November 24, 2019) revealed acute dermatitis and cellulitis associated with fungal infection. Fungal spores and other fungal structures were seen in follicular infundibulae and in the superficial cornified layer. These were positive for the fungal cell wall stains periodic acid schiff (PAS) and Grocott methenamine silver. Neutrophilic exocytosis in the superficial epidermis, rarefaction of sebaceous glands with destruction of the deep portion of hair follicles, severe diffuse neutrophilic inflammatory infiltrate in the reticular dermis and subcutaneous tissue, tissue necrosis and phagocytosis of cell debris by sparse multinucleated histiocytes were also observed.

Fungal culture results obtained on November 26, 2019 revealed *Trichophyton mentagrophytes*. Hence, fluconazole was replaced with itraconazole and griseofulvin maintained.

**Figure 2:**
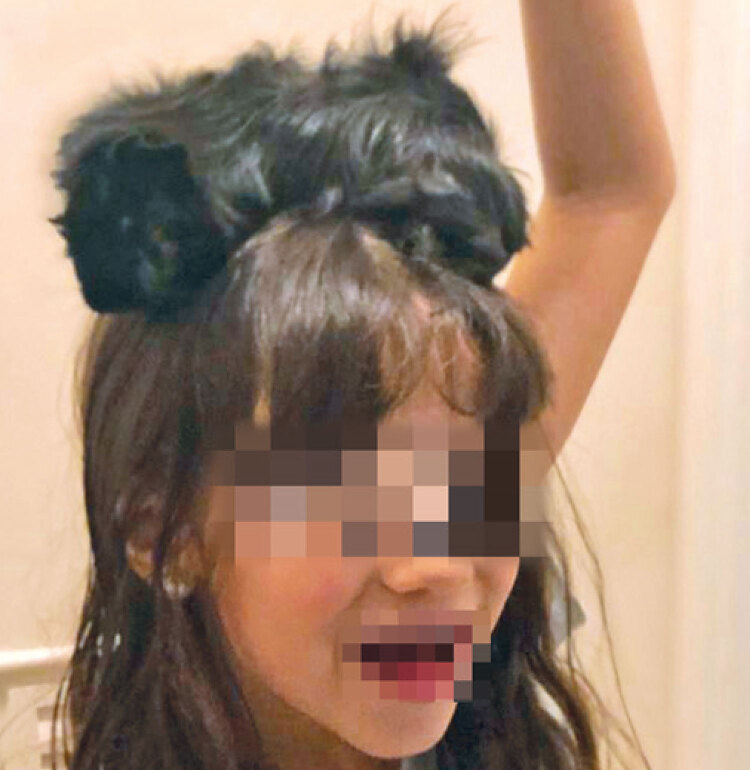
Patient with the guinea pig sitting on top of her head

Given the final diagnosis, parents were asked about contact with animals and reported having a guinea pig. A picture showing the child with the guinea pig sitting on top of her head was provided (Figure 2).

Wound debridement was repeated and dressings changed three times over the course of hospital stay. Scalp swelling and inflammation gradually subsided.

**Figure 3:**
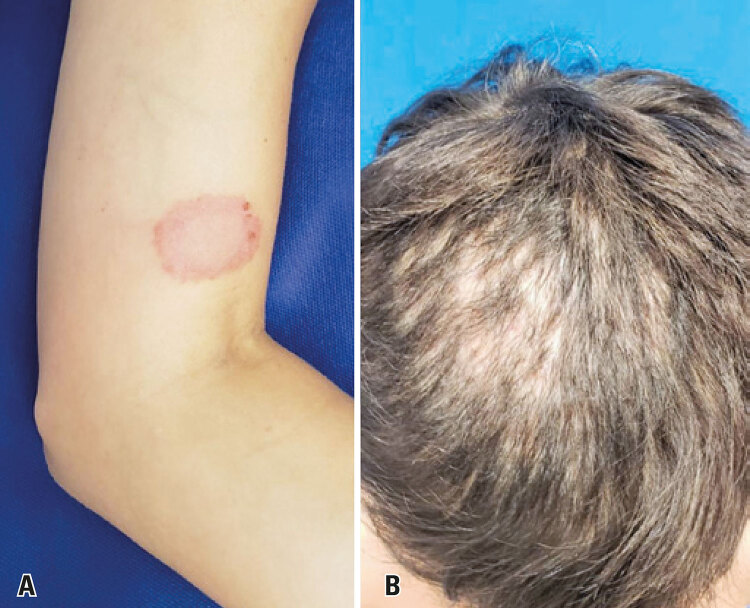
(A) Lesion on the mother’s left upper limb; (B) Patient scalp within 3 months of hospital admission

Parents were questioned regarding similar lesions in family members. The child’s mother had a single 5cm-wide lesion on the left upper limb. Direct examination results were negative. Therefore, biopsy was collected. Histologic examination revealed dermatophytosis. Fungal culture confirmed *Trichophyton mentagrophytes* (Figure 3A).

The patient improved significantly and was discharged from hospital within 9 days of admission. Surgical procedures were repeated at later dates (December 2, 6 and 13, 2019).

Overall, the patient received sodium cefuroxime for 9 days, itraconazole combined with griseofulvin for two weeks, then griseofulvin alone for another six weeks. Antimicrobial therapy was prescribed due to potential secondary bacterial infection.

Partial healing of scalp lesions and alopecia was achieved in the mid term. However, full recovery of hair follicles could not be attained (Figure 3B).

This study was approved by the Research Ethics Committee of *Hospital Israelita Albert Einstein* under # 4.574.726, CAAE: 44044221.5.0000.0071.

## DISCUSSION

Dermatophytosis is a zoonotic disease transmitted to humans through direct contact with animals and a highly prevalent infection in Latin America. One or more reddish, limited sized and potentially coalescing circular lesions may develop in infected skin. Lesions may be found anywhere in the body. However, the scalp is often affected.^(8)^

Dermatophyte infections may range from mild to severe, depending of host reaction to metabolites produced by the fungus, virulence of the infecting species, anatomical location and environmental factors.^(9,10)^

Fumeaux et al. isolated *Arthroderma benhamiae*, a species of the *Trichophyton mentagrophytes* complex, from nine individuals (one eight-year-old child, seven adolescents and one 30-year-old adult) with inflammatory dermatophytosis. Of these, eight had a history of prior contact with rodents, particularly guinea pigs.^(11)^

In a study published in 2013, *Trichophyton mentagrophytes* was isolated from 19 out of 20 asymptomatic guinea pigs housed in a zoo in the city of Urayasu, Japan.^(12)^

In 2019, Bartosch et al. demonstrated that, in a group of 41 animals housed together (rabbits, rats, mice and guinea pigs), 18 out of 26 guinea pigs had dermatophytosis, although only 11 showed clinical signs. Of 18 confirmed cases, 11 were caused by *Trichophyton mentagrophytes*. Dermatophytosis was less common in the other animals and rabbits were not affected.^(13)^

Most guinea pigs infected with *Trichophyton mentagrophytes* fail to develop clinical signs and become asymptomatic carriers of the fungus. The prevalence of *Trichophyton mentagrophytes* infection ranges from 1.4 to 34.9% in these animals.^(8,11)^

Systemic antifungal treatment is thought to be the gold standard in children with *Tinea capitis*. Griseofulvin and terbinafine (first line and short course treatment respectively) are the two drugs approved by the FDA (U.S. Food and Drug Administration) for use in pediatric patients. Although controversial, treatment with systemic corticosteroids may be indicated in cases of *Kerion celsi* with significant inflammation. Importantly, alopecia may persist for life in some cases.^(14)^

This case report is a wake-up call for potential disease transmission by pet rodents. The significance of comprehensive medical history taking to investigate patient household environment characteristics is emphasized.
